# Isotope analysis reveals mobility of the earliest cattle herds in northern Europe

**DOI:** 10.1038/s41598-026-66057-4

**Published:** 2026-09-06

**Authors:** Nathalie Ø. Brusgaard, Lisette M. Kootker, Daan C. M. Raemaekers

**Affiliations:** 1 Faculty of Archaeology, Leiden University, Einsteinweg 2, Leiden, 2333 CC the Netherlands; 2 Faculty of Science, Department of Earth Sciences, Geology & Geochemistry cluster, Vrije Universiteit Amsterdam, de Boelelaan 1100, Amsterdam, 1081 HZ the Netherlands; 3 Faculty of Social Sciences and Humanities, Department of Art and Culture, History, and Antiquity, Antiquity cluster, Vrije Universiteit Amsterdam, de Boelelaan 1105, Amsterdam, 1081 HV the Netherlands; 4 Groningen Institute of Archaeology, University of Groningen, Postbus 716, Groningen, 9700 AS the Netherlands

**Keywords:** Neolithic, Neolithisation, Livestock husbandry, Mobility, Isotope analysis, Biochemistry, Archaeology

## Abstract

Human mobility was intrinsic to the farming communities of the Linear Pottery culture (c. 5500 − 4900 BCE). Much less is known about the mobility patterns of the earliest farmers north of the Linear Pottery zone, particularly in the Netherlands. Here, people of the Early Neolithic Swifterbant culture combined farming, hunting and gathering from c. 4240 BCE. While human remains for mobility studies are scarce, livestock offer a promising yet underexplored proxy. This research applies inter- and intra-tooth strontium (^87^Sr/^86^Sr), oxygen (*δ*^18^O), and carbon (*δ*^13^C) isotope analysis to cattle, suids, and sheep from the Swifterbant sites S3 and S4. Previous isotope research identified two distinct livestock dietary regimes: animals grazing in floodplain environments and animals grazing in forested settings. The results presented here reveal substantial variability in livestock mobility, including several non-local animals that moved multiple times during their lifetime. Notably, animals associated with the forest-grazing dietary regime display non-local strontium isotope values, whereas individuals linked to floodplain-grazing are predominantly local. These findings provide the first evidence of livestock mobility in northern Europe during the fifth millennium BCE and suggest that the movement of animals was part of broader interaction networks among early farming communities. The study further demonstrates that, in addition to intra-individual isotope variation, intra-population variability in livestock can reveal patterns of mobility and animal management.

**Supplementary Information:**

The online version contains supplementary material available at https://doi.org/10.1038/s41598-026-66057-4.

## Introduction

In the debate on the Neolithisation of Europe, mobility is often discussed in one of two contexts: the mobility of indigenous foragers – juxtaposed to settled farmers – and the migration of farmers beyond the agricultural frontier into new territories. It is generally accepted that the latter led to the swift spread of the earliest farmers in Europe, the Linear Pottery culture (LBK, c. 5500 − 4900 BCE), although likely in a ‘leap-frog’ manner rather than as a wave of migration^1–5^. Mobility was intrinsic to the LBK, not just in the form of migratory colonisation but also everyday mobility between settlements and regions^2,6^. Farmers were therefore not necessarily ‘settled’ once established somewhere^3,7^.

Owing to gaps in current knowledge, the mobility of the late foragers and earliest farmers living north of the LBK zone is less well defined. Farming emerged in this region towards the end of the 5th millennium BCE, with recent evidence pointing to a transition date of 4240 BCE in the Netherlands^8–10^ and around 4000 BCE in Britain and southern Scandinavia^11,12^. The emergence of agriculture in this region was paired with the exchange of objects, ideas, and genetic material among populations^1,13–16^. The latter and a limited number of isotope studies currently inform knowledge on the movement of people in this region. There is indirect yet convincing isotopic evidence for mobility of Ertebølle foragers in Denmark^17^. Isotope analysis of Ertebølle communities in Skateholm, Sweden, identified no non-local individuals, although this need not mean an absence of mobility^18^. Isotope data yielded limited evidence for mobility among people from the Swifterbant culture in the Netherlands^19^. Recent genetic evidence for the integration of women with Early European Farmers ancestry into Swifterbant hunter-gatherer communities raises the possibility that their movement was connected to the spread of knowledge of farming^20^.

The nature of the mobility of people at and above the agricultural frontier can thus provide insight into how farming spread in northern Europe. It can also inform on how forager-farmer groups in the Lower Rhine Basin (Swifterbant culture) were organised and when they had transitioned to farming. Hampering mobility studies is the fact that well-preserved human remains from this period are relatively scarce. Other proxies for mobility are indirect by nature. These comprise evidence of flooding events during the occupation of the levees, suggesting seasonal habitation by Swifterbant people^21^, the on-site presence of flint and other stone material in a natural environment where stones are absent^22^, and hunting and fishing patterns that are seasonal^23^. However, another proxy for mobility has remained largely unstudied from this region: livestock. The movement of livestock requires, at least to some degree, the movement of people. Myriad isotope studies have demonstrated the value of studying the mobility of domesticated animals to gain insight into the mobility of humans, the extent of exchange networks, and the nature of human-animal relationships^24–28^.

To advance the understanding of the movements of early farmers and the debate on the agricultural transition in northern Europe, this paper presents a combined strontium (^87^Sr/^86^Sr), oxygen (*δ*^18^O), and carbon (*δ*^13^C) isotope analysis of cattle, suids, and sheep from the Swifterbant culture sites S3 and S4 in the Netherlands (c. 4240 − 4050 BCE). These assemblages provide a uniquely valuable dataset for exploring mobility within the Swifterbant culture. The cattle constitute the earliest known domestic cattle in northern Europe, and previous dietary isotope analysis revealed two distinct dietary groups among them, opening the possibility that one or more animals came from elsewhere^10^. The occurrence of different livestock dietary regimes within one settlement is rare in prehistoric Europe. It has been documented only twice in Neolithic Europe, at a site in Iberia^29^ and possibly at a Dutch site^30^, and once in Bronze Age Turkey^31^. It raises important questions regarding prehistoric livestock management, including the possibility that the herds were imported from different regions. If confirmed, the Swifterbant case would represent the earliest evidence of livestock mobility in northern Europe. Currently, the earliest documented examples are from the later Funnel Beaker Culture (TRB), dating to after 4000 BCE^32,33^. As the first mobility study on Dutch Neolithic animals and only the second on prehistoric animals in this region, this research furthermore provides an important contribution to the understanding of early animal mobility in northern Europe.

The primary aim of this study is to study livestock mobility at a population level and assess whether either of the two dietary groups included animals originating from outside the Swifterbant region. To this end, strontium and oxygen isotope analyses were conducted on all suitable cattle (*n* = 22) and sheep (*n* = 2) teeth and a selection of suid teeth from domestic individuals (*n* = 6). Each cattle and sheep tooth was sampled at two to three positions along the enamel growth axis to maximize the likelihood of detecting temporal changes in isotopic composition indicative of mobility. A secondary aim is to further characterize livestock diet through carbon isotope analysis.

### Background

**Fig. 1:**
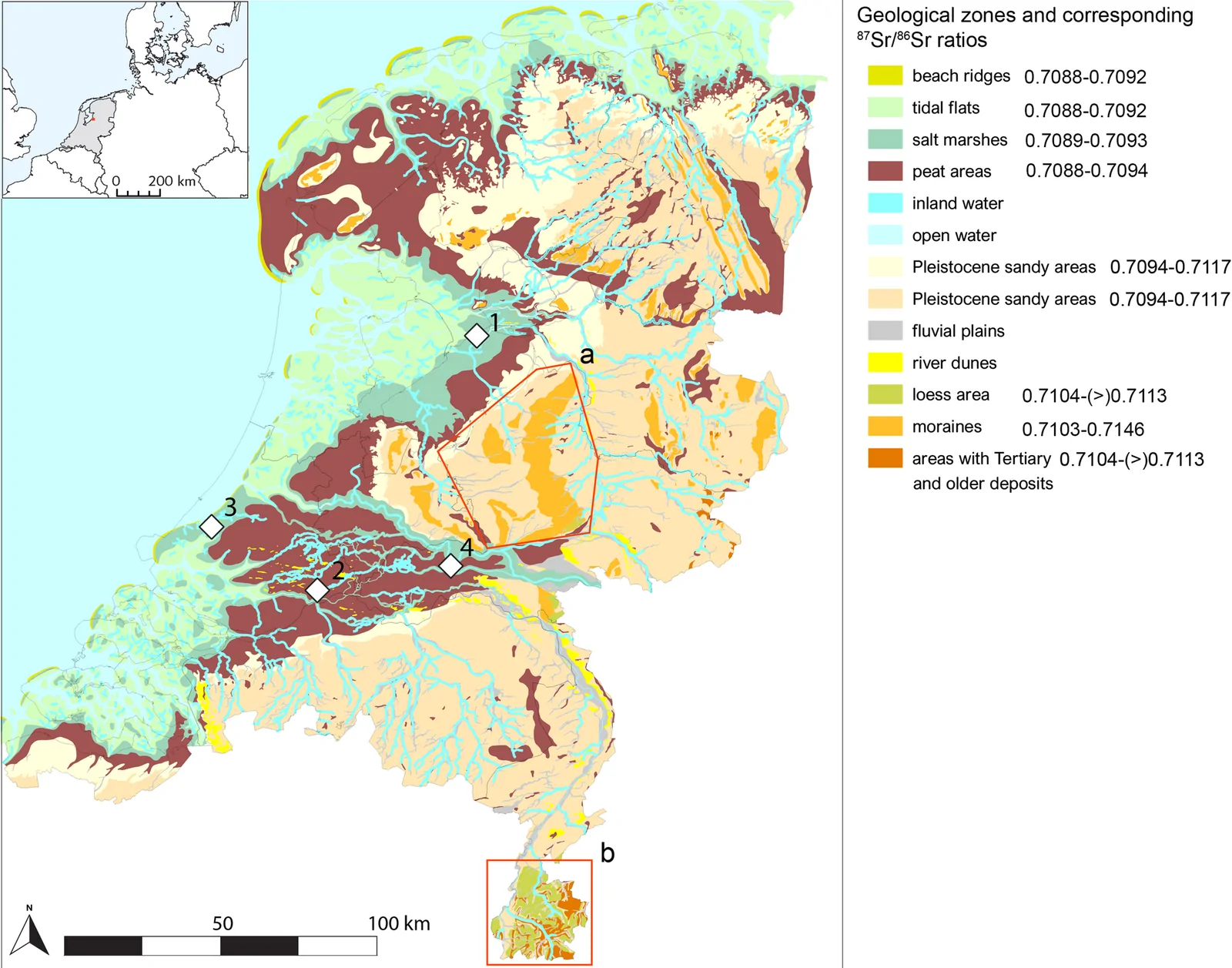
Palaeogeographic map of the Netherlands c. 3850 BCE. Location of Neolithic sites mentioned in the text: (1) Swifterbant S2, S3, and S4, (2) Hardinxveld-Giessendam Polderweg and De Bruin, (3) Schipluiden, (4) Tiel-Medel. Location of regions mentioned in the text: (a) Veluwe, (b) southern Limburg. Base map by Vos et al.^91^ ^87^Sr/^86^Sr ranges based on Kootker et al.^58^ and De Coster et al.^88^.

The Swifterbant culture has been identified in the Netherlands, Belgium, and Germany from c. 5000 BCE onwards^34,35^. The oldest sites are Late Mesolithic Hardinxveld-Giessendam Polderweg and De Bruin (5500 − 4250 BCE) in the Netherlands (Fig. 1). De Bruin has the oldest Swifterbant culture pottery, dated to 5000 BCE, and around this time this site shows indications of changing from being a seasonal hunting-gathering camp to having more year-round occupation^36–38^. This does not, however, appear to be part of a clear-cut transition to a settled existence. Repeated flooding of the levees at the Early Neolithic S3 and S4 sites (4250 − 4050 BCE), suggests that people did not occupy these settlements year-round^39^.

While diverse wild resources in this freshwater environment remained important during the Early Neolithic^40,41^, the earliest evidence for animal management and crop husbandry already appears around 4240 BCE, uncovered at the S3 and S4 sites^10,21,39,42^. Livestock keeping is evidenced by the dietary changes among the animals, their small stature, the presence of cranial and postcranial skeletal elements suggesting on-site slaughtering, a foetal cattle bone suggesting cattle breeding, and pig coprolites containing sheep’s wool and cereal grains^10,40,43^. The omni-presence of evidence for cereal cultivation and consumption testifies to the structural role this new food category played in the daily lives of the Swifterbant culture^21,39,42–44^. There are also indications for farming among Swifterbant culture sites in Belgium, but exactly how early farming started in this region is still a matter of debate^45–48^.

By the end of the fifth millennium BCE the Swifterbant culture had thus integrated crop cultivation and animal husbandry with a foraging subsistence. Animal husbandry included clear management of livestock diet, as evidenced by the S3 and S4 sites. Here one group of livestock has high *δ*^15^N values while the other has low *δ*^13^C values, raising the possibility that one or even both groups may have come from elsewhere^10^. The first group, consisting of 30 cattle (*Bos taurus* based on size), has mean *δ*^15^N values of 11.6 ± 1.3‰. Three sheep (*Ovis aries*) have similarly high *δ*^15^N values (mean 12.6 ± 0.8‰). Equally high *δ*^15^N values in herbivores have thus far only been observed in animals grazing in salt marshes or on manured pasturelands^49–51^. This was proposed as a potential explanation for the *δ*^15^N values observed in this group^10^. However, a recent landscape-focused isotope study demonstrated that in the Netherlands, regular flooding of estuarine wetland areas can substantially elevate *δ*¹⁵N values in local soils, thereby affecting the isotopic signatures of plants and the herbivores that consume them^52^. The Swifterbant levees were subject to regular flooding from the river system^39^. At S4, which spans approximately 90 years^8^, several such flooding events have been documented. It is therefore highly likely that the higher *δ*^15^N values in this group of Swifterbant cattle and sheep reflects local grazing in a floodplain context. The *δ*^13^C_col_ values of these cattle (mean − 21.1 ± 0.3‰) and sheep (mean − 21.5 ± 0.3‰) are congruent with grazing on C_3_ plants in an open landscape^53,54^.

The second group, consisting of 12 cattle (based on size, *Bos taurus* except one *Bos* sp.), has *δ*^15^N values (mean 5.6 ± 0.7‰) equivalent to those expected in herbivores, and lower *δ*^13^C_col_ values (mean − 23.1 ± 0.5‰). One caprine (*Ovis aries*/*Capra hircus*) has similar *δ*^13^C_col_ values (–22.4‰) and a slightly higher *δ*^15^N values (8.25‰). The *δ*^13^C_col_ values of these animals are congruent with grazing consistently on C_3_ plants growing in a forested environment, under influence of the canopy effect^10,53–55^.

Three small-sized suids (*Sus* sp.; *Sus domesticus*/*Sus scrofa*) at the sites also had significantly high *δ*^15^N values (> 9‰), suggesting they were domestic pigs (*Sus domesticus*) that foraged on human refuse, or foraged in the same environment as the first group of livestock^10,56^.

The significant difference in diets between the two groups suggests that one group was pastured elsewhere. Given the expected slow turnover rate of cortical bone collagen in fully grown individuals, it is plausible that the adult animals could have spent a considerable period at Swifterbant before slaughter, while still retaining the dietary signal from their place of origin^57^.

## Results

**Fig. 2:**
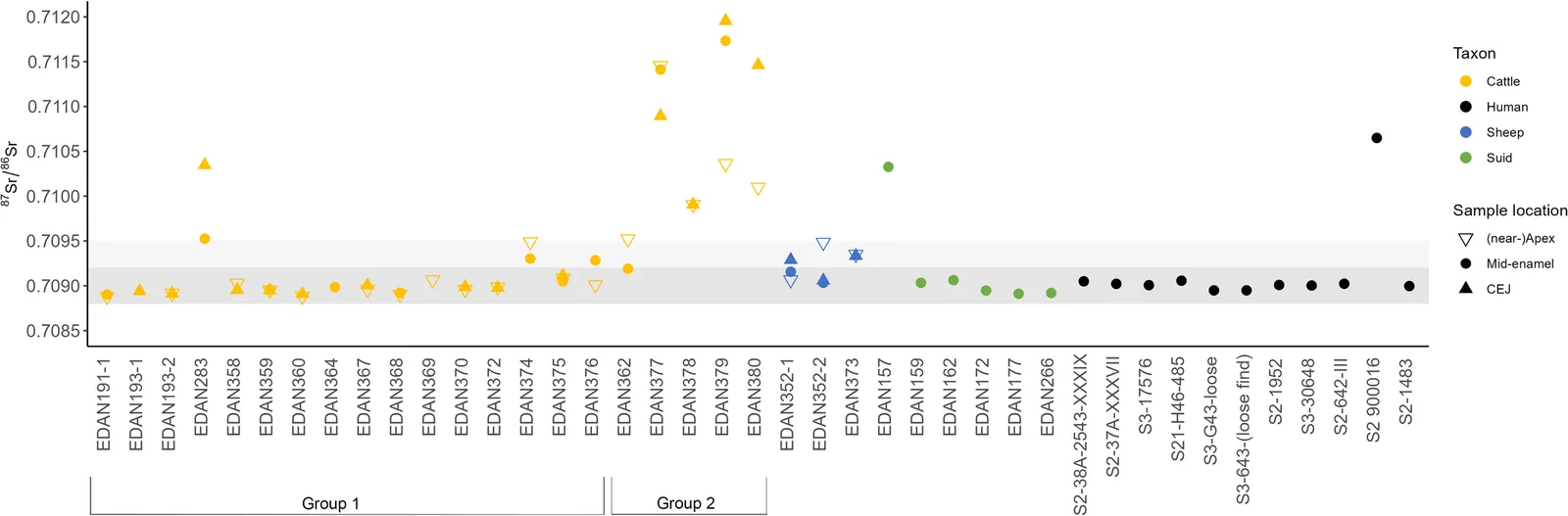
Univariate plot showing ^87^Sr/^86^Sr results of Swifterbant cattle, sheep, and suids, compared to previously published Swifterbant human data^19^. Background shading indicates the local ^87^Sr/^86^Sr range (dark grey: 0.7088–0.7092) and the regional ^87^Sr/^86^Sr range (light grey: 0.7092–0.7095). Group 1 and Group 2 refer to the two dietary groups among the cattle (see Background).

**Fig. 3:**
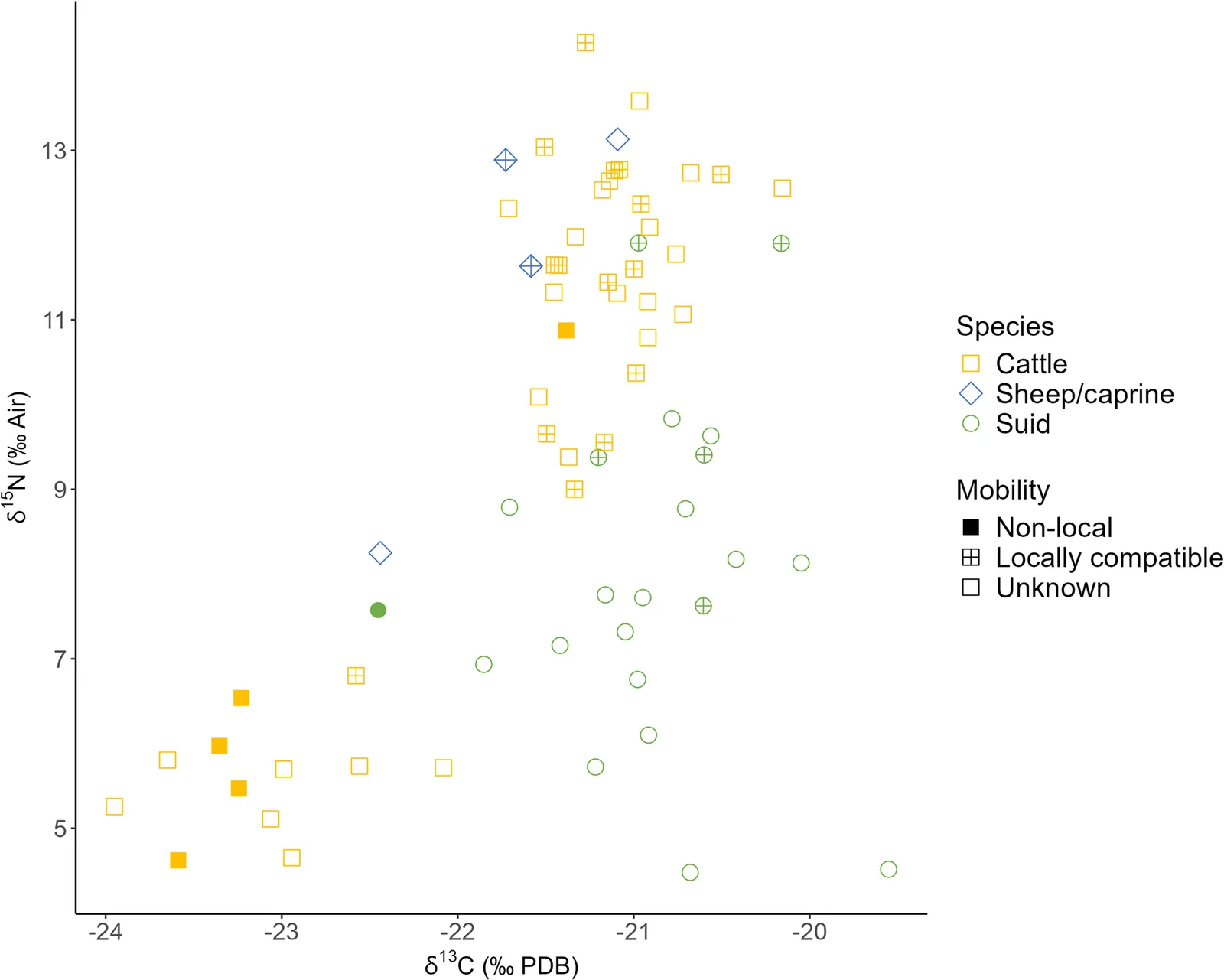
Bivariate plot showing *δ*^13^C and *δ*^15^N data of the Swifterbant cattle, sheep, and suids compared to their mobility based on the ^87^Sr/^86^Sr results. Non-local = individual has ^87^Sr/^86^Sr values incompatible with the local ^87^Sr/^86^Sr range. Locally compatible = individual has ^87^Sr/^86^Sr values compatible with the local range. Unknown = no ^87^Sr/^86^Sr were obtained for the individual. *δ*^13^C and *δ*^15^N data from previously published study^10^.

**Fig. 4:**
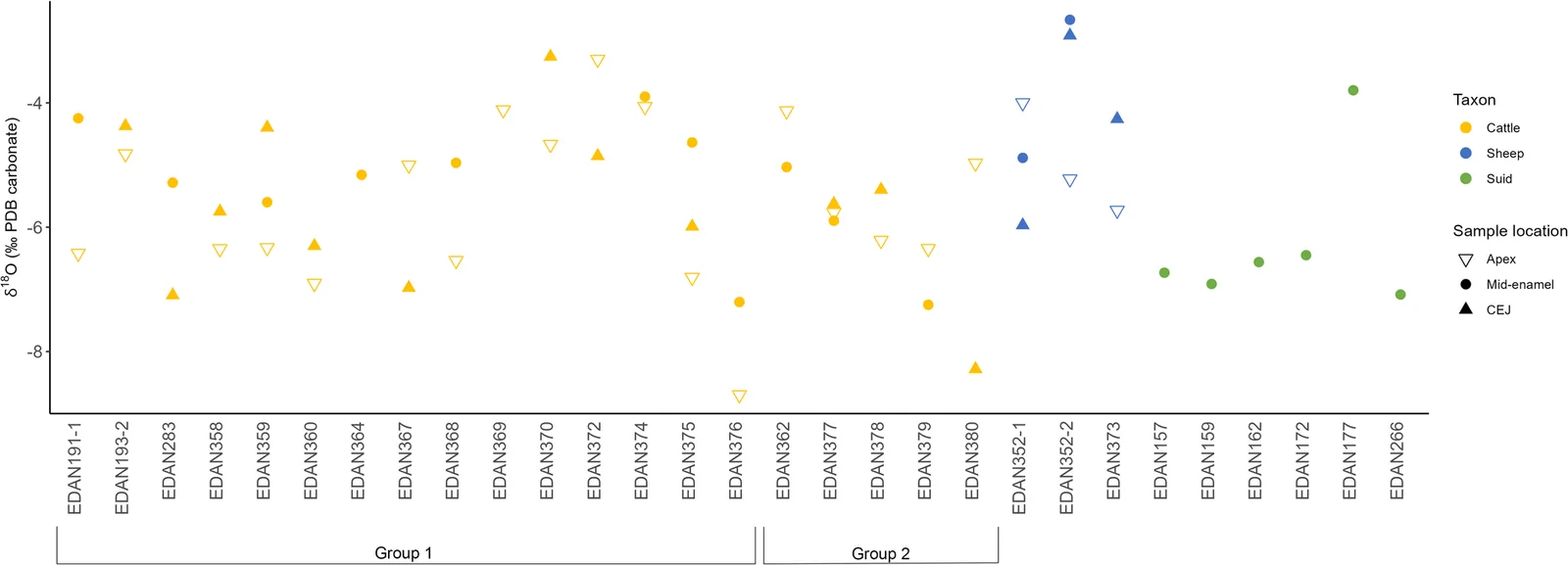
Univariate plot showing *δ*^18^O results of Swifterbant cattle, sheep, and suids.

**Fig. 5:**
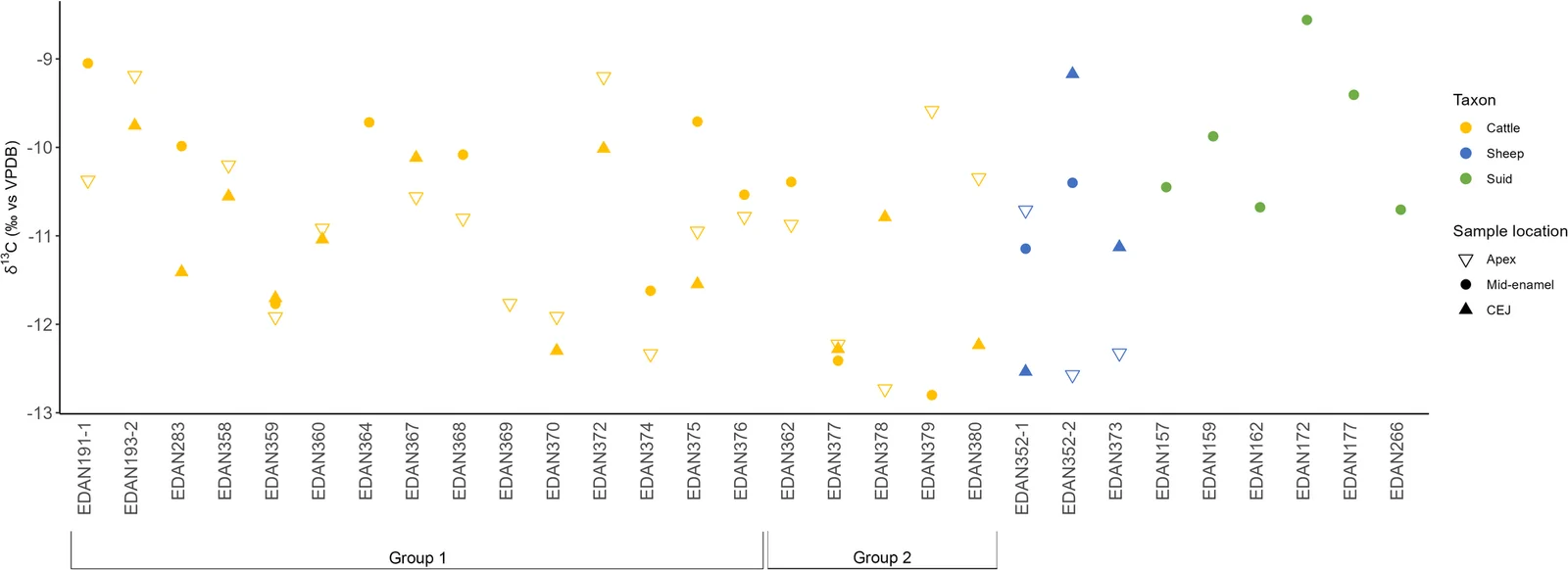
Univariate plot showing *δ*^13^C results of Swifterbant cattle, sheep, and suids.

The results are presented in Tables 1 and 2; Figs. 2, 3, 4 and 5. A total of 57 ^87^Sr/^86^Sr and 55 *δ*^13^C_PDB_ and *δ*^18^O_PDB_ were determined, representing 28 unique individuals (Table 1). The ^87^Sr/^86^Sr range from 0.708881 to 0.711955 (^87^Sr/^86^Sr_max−min_ = 0.003074). Intra-dental variation in ^87^Sr/^86^Sr is generally limited in most cattle (< 0.0003), except for individuals EDAN283, EDAN377, EDAN379 and EDAN380, which display substantially greater variation (> 0.0005). *δ*^13^C_PDB_ values range from − 12.8‰ to − 8.6‰ (range = 4.2), and *δ*^18^O_PDB_ values vary between − 8.7‰ and − 2.7‰ (range = 6.0). Intra-dental variation in ^87^Sr/^86^Sr was also limited in the sheep (three teeth from two individuals), with all within-tooth differences < 0.0004. Compared to cattle, sheep exhibited a narrower overall range in both carbon and oxygen isotope compositions, with *δ*^13^C_PDB_ values ranging from − 12.5‰ to − 9.2‰ and *δ*^18^O_PDB_ values from − 6.0‰ to − 2.7‰ (range = 3.3‰ for both isotope systems). A considerable spread is observed in the suid ^87^Sr/^86^Sr data, which range from 0.708911 to 0.710326 (^87^Sr/^86^Sr_max−min_ = 0.001415). In contrast, the ranges in *δ*^13^C_PDB_ and *δ*^18^O_PDB_ are comparable to, or narrower than, those observed in the other two species (2.1‰ and 3.3‰, respectively; Table 2).

**Table 1:** Sr-O-C-N isotope data from Swifterbant culture sites S3 and S4 in the Netherlands. The within run precisions (2SE) for Sr were consistently ≤ 0.000010. The stable isotope data are expressed in ‰. Key: CEJ – cemento-enamel junction; M – molar; M1 – first molar; M2 – second molar; M3 – third molar; dP4 – deciduous fourth premolar; DPB – Pee Dee Belemnite; col – collagen; * - previously published data^10^. All data are available on IsoArch.org.

Site	Toponym	Taxon	Element	Sample location	Sample ID	^87^Sr/^86^Sr	*δ*^13^C_PDB_	*δ*^18^O_PDB_	*δ*^13^C_col_*	*δ*^15^N_col_*
Swifterbant	S3	*Bos taurus*	M1	Middle	EDAN191-1-2	0.708903	−9.0	-4.2	–	–
				Apex	EDAN191-1-3	0.708881	−10.4	−6.4	−21.1	11.4
Swifterbant	S3	*Bos taurus*	M1	CEJ	EDAN193-1-1	0.708940	–	–	–	–
Swifterbant	S3	*Bos taurus*	M2	CEJ	EDAN193-2-1	0.708913	−9.8	−4.4	–	–
				Apex	EDAN193-2-3	0.708917	−9.2	−4.8	−21.1	12.8
Swifterbant	S3	*Bos taurus*	M3	CEJ	EDAN283-1	0.710347	−11.4	−7.1	−21.4	10.9
				Middle	EDAN283-2	0.709525	−10.0	−5.3	–	–
Swifterbant	S4	*Bos taurus*	M	CEJ	EDAN358-1	0.708954	−10.6	−5.7	–	–
				Apex	EDAN358-3	0.709029	−10.2	−6.3	−21.0	11.6
Swifterbant	S4	*Bos taurus*	dP4	CEJ	EDAN359-1	0.708948	−11.7	−4.4	–	–
				Middle	EDAN359-2	0.708955	−11.8	−5.6	–	–
				Apex	EDAN359-3	0.708953	−11.9	−6.3	−21.0	12.4
Swifterbant	S4	*Bos taurus*	M2/M3	CEJ	EDAN360-1	0.708910	−11.0	−6.3	–	–
				Apex	EDAN360-3	0.708882	−10.9	−6.9	−21.1	12.8
Swifterbant	S3	*Bos taurus*	M1/M2	Middle	EDAN362-2	0.709190	−10.4	−5.0	–	–
				Apex	EDAN362-3	0.709523	−10.9	−4.1	−22.6	6.8
Swifterbant	S3	*Bos taurus*	M2	Middle	EDAN364-2	0.708985	−9.7	−5.2	–	–
Swifterbant	S3	*Bos taurus*	M1	CEJ	EDAN367-1	0.709005	−10.1	−7.0	–	–
Swifterbant	S3	*Bos taurus*	M1	Apex	EDAN367-3	0.708959	−10.6	−5.0	−21.3	9.0
Swifterbant	S3	*Bos taurus*	M2	Middle	EDAN368-2	0.708922	−10.1	−5.0	–	–
				Apex	EDAN368-3	0.708905	−10.8	−6.5	−20.5	12.7
Swifterbant	S3	*Bos taurus*	M1/2	Apex	EDAN369-3	0.709066	−11.8	−4.1	−21.3	14.3
Swifterbant	S3	*Bos taurus*	dP4	CEJ	EDAN370-1	0.708986	−12.3	−3.3	–	–
				Apex	EDAN370-3	0.708959	−11.9	−4.7	−21.5	13.0
Swifterbant	S3	*Bos taurus*	M3	CEJ	EDAN372-1	0.708978	−10.0	−4.9	–	–
				Apex	EDAN372-3	0.708986	−9.2	−3.3	−21.0	10.4
Swifterbant	S3	*Bos taurus*	M1/M2	Middle	EDAN374-2	0.709303	−11.6	−3.9	–	–
				Apex	EDAN374-3	0.709490	−12.3	−4.1	−21.2	9.6
Swifterbant	S3	*Bos taurus*	M3	CEJ	EDAN375-1	0.709114	−11.5	−6.0	–	–
				Middle	EDAN375-2	0.709050	−9.7	−4.6	–	–
				Apex	EDAN375-3	0.709088	−10.9	−6.8	−21.5	9.7
Swifterbant	S3	*Bos taurus*	M3	Middle	EDAN376-2	0.709284	−10.5	−7.2	–	–
				Apex	EDAN376-3	0.709010	−10.8	−8.7	−21.5	11.6
Swifterbant	S3	*Bos taurus*	M1/M2	CEJ	EDAN377-1	0.710893	−12.3	−5.6	–	–
				Middle	EDAN377-2	0.711413	−12.4	−5.9	–	–
				Apex	EDAN377-3	0.711456	−12.2	−5.8	−23.6	4.6
Swifterbant	S3	*Bos taurus*	M1/M2	CEJ	EDAN378-1	0.709906	−10.8	−5.4	–	–
				Apex	EDAN378-3	0.709907	−12.7	−6.2	−23.2	5.5
Swifterbant	S3	*Bos taurus*	M2	CEJ	EDAN379-1	0.711955	–	–	–	–
				Middle	EDAN379-2	0.711733	−12.8	−7.2	–	–
				Apex	EDAN379-3	0.710362	−9.6	−6.3	−23.2	6.5
Swifterbant	S3	*Bos taurus*	M3	CEJ	EDAN380-1	0.711463	−12.2	−8.3	–	–
				Apex	EDAN380-3	0.710099	−10.3	−5.0	−23.4	6.0
Swifterbant	S3	*Ovis aries*	M1	CEJ	EDAN352-1-1	0.709289	−12.5	−6.0	–	–
				Middle	EDAN352-1-2	0.709157	−11.1	−4.9	–	–
				Apex	EDAN352-1-3	0.709066	−10.7	−4.0	–	–
			M2	CEJ	EDAN352-2-1	0.709060	−9.2	−2.9	–	–
				Middle	EDAN352-2-2	0.709034	−10.4	−2.7	–	–
				Apex	EDAN352-2-3	0.709484	−12.6	−5.2	−21.6	11.6
Swifterbant	S3	*Ovis aries*	M1/M2	CEJ	EDAN373-1	0.709334	−11.1	−4.3	–	–
				Apex	EDAN373-3	0.709350	−12.3	−5.7	−21.7	12.9
Swifterbant	S3	*Sus scrofa/domesticus*	M2	Middle	EDAN157-1	0.710326	−10.4	−6.7	−22.5	7.6
Swifterbant	S3	*Sus scrofa/domesticus*	M2	Middle	EDAN159-1	0.709033	−9.9	−6.9	−21.2	9.4
Swifterbant	S3	*Sus scrofa/domesticus*	M1	Middle	EDAN162-1	0.709063	−10.7	−6.6	−20.2	11.9
Swifterbant	S3	*Sus scrofa/domesticus*	M2	Middle	EDAN172-1	0.708947	−8.6	−6.5	−20.6	7.6
Swifterbant	S3	*Sus scrofa/domesticus*	M1	Middle	EDAN177-1	0.708911	−9.4	−3.8	−20.6	9.4
Swifterbant	S3	*Sus scrofa/domesticus*	P4	Middle	EDAN266-1	0.708920	−10.7	−7.1	−21.0	11.9

**Table 2:** Summary table of the ^87^Sr/^86^Sr, *δ*^13^C_PDB_ and *δ*^18^O_PDB_ results per group and taxon.

Group/species	^87^Sr/^86^Sr range	^87^Sr/^86^Sr mean	^87^Sr/^86^Sr n of samples	*δ*^13^C_PDB_ range	*δ*^13^C_PDB_ mean	*δ*^13^C_PDB_ n of samples	*δ*^18^O_PDB_ range	*δ*^18^O_PDB_ mean	*δ*^18^O_PDB_ n of samples
Group 1 *Bos*	0.708881–0.710347	0.709230 ± 0.000735	30	−12.3 to −9.0‰	−11.0 ± 1.0‰	29	−8.7 to −3.3‰	−5.5 ± 1.3‰	29
Group 2 *Bos*	0.709289 - 0.711955	0.710532 ± 0.001072	10	−12.8 to −9.6‰	−11.6 ± 1.2‰	9	−8.3 to −4.1‰	−6.1 ± 1.3‰	9
*Ovis aries*	0.709034–0.709484	0.709222 ± 0.000166	8	−12.6 to −9.2‰	−11.2 ± 1.2‰	8	−6.0 to −2.7‰	−4.5 ± 1.2‰	8
*Sus scrofa/domesticus*	0.708911–0.710326	0.709200 ± 0.0.000555	6	−10.7 to −8.6‰	−9.9 ± 0.8‰	6	−7.1 to −3.8‰	−6.3 ± 1.2‰	6

The group 1 cattle that likely grazed in the local floodplains display ^87^Sr/^86^Sr between 0.708881 and 0.710347 (Table 2, Table S1). Their *δ*^13^C_PDB_ and *δ*^18^O_PDB_ values range respectively from − 12.3‰ to − 9.0‰ and from − 8.7‰ to − 3.3‰. The second group, interpreted as grazing in more forested environments, shows a narrower isotopic range, with similar *δ*^13^C_PDB_ and *δ*^18^O_PDB_ values, but significant higher mean strontium isotope ratios. Their ^87^Sr/^86^Sr vary between 0.709190 and 0.711955, while *δ*^13^C_PDB_ and *δ*^18^O_PDB_ values range from − 12.8‰ to − 9.6‰ and from − 8.3‰ to − 4.1‰ respectively (Fig. 1).

## Discussion

### Mobility and diet among the Early Neolithic livestock

The strontium isotope data provide evidence for the presence of non-local livestock (^87^Sr/^86^Sr > 0.7095), specifically five cattle (EDAN283, 377, 378, 379, and 380) and one pig (EDAN157) at Swifterbant (Fig. 2; Table 1). Moreover, the data suggest that almost all animals that – based on their dietary isotope signatures – grazed in forested environments, originated from outside the region (Fig. 3). Five cattle from this group (Group 2) (mean *δ*¹³C_col_ = − 23.1 ± 0.5‰), were sampled, and four of these are now confirmed as non-local. Notably, the single non-local pig (EDAN157) exhibits similarly low *δ*¹³C_col_ values, aligning it isotopically with this group. In contrast, within the group displaying high *δ*¹⁵N_col_ values (mean = 11.6 ± 1.3‰), interpreted as reflecting floodplain-grazing, only one individual (EDAN283) is identified as non-local. It should be emphasized, however, that the ^87^Sr/^86^Sr baseline observed at Swifterbant is also found across large parts of the western Netherlands^58^, meaning that animals that exhibit Sr isotope ratios compatible to the region (c. 0.7088–0.7095) may still have originated elsewhere.

Assuming that intra-dental shifts in ^87^Sr/^86^Sr in cattle are the result of mobility rather than dietary changes (e.g., provisioned fodder), the significant intra-dental variation observed in four cattle indicates multiple moves during their early years of life (Figs. S1 and 2). For example, cattle EDAN379 shows evidence for having moved twice between the age of ca. 2 and 12 months old (the age of mineralisation of the M2, Table 3). The patterns of lifetime mobility also vary among these individuals: while EDAN379 and 380 moved from areas with lower to higher ^87^Sr/^86^Sr, EDAN283 and 377 show the opposite trend, having moved from a region with more radiogenic to less radiogenic strontium ratios. This reinforces the interpretation that these animals were imported to Swifterbant and points to variability in their geographic origins.

**Table 3:** The age of enamel mineralisation for cattle and sheep molars.

Dental element	Age of enamel mineralisation	
	Cattle^92^	Sheep^93^
Fourth deciduous premolar (dP4)	–	–
First molar (M1)	0–3 months	2 months prior to birth − 6 months
Second molar (M2)	2–12 months	1–12 months
Third molar (M3)	9–24 months	10–22 months

The question arises whether the *δ*^18^O data from Swifterbant provide additional, independent evidence for mobility, perhaps even among individuals whose ^87^Sr/^86^Sr are consistent with the local baseline. The non-local group 2 cattle have a range of − 4.1‰ to − 8.3, while the group 1 cattle have an even wider range of − 3.3‰ to − 8.7‰ (Fig. 4). One sheep (EDAN352) displays a comparatively high *δ*¹⁸O value of − 2.7‰. Some *δ*^18^O variability may reflect physiological factors such as suckling, given that breastmilk is enriched in *δ*^18^O^59^. For instance, EDAN352 was obtained from an M2, which mineralises between 1 and 12 months of age (Table 3). The M2 therefore reflects a period during which suckling may still occur. However, this does not fully explain the observed range. The highest *δ*^18^O value recorded for cattle (–3.3‰) derives from an M3 (EDAN372; near apex sample), which mineralises between 9 and 24 months and is unlikely to retain a nursing signal. Modern feral and semi-wild herds of cattle wean their calves naturally between a few months and 12 months old, the average age being around 8 months^25,60^. Studies of Neolithic cattle populations suggest that artificial weaning sometimes occurred at an earlier age^61,62^. These observations suggest that, at least in some cases, *δ*¹⁸O variation reflects something other than solely metabolic effects.

For comparison, the *δ*^18^O range in the modern, semi-feral Chillingham cattle population from Northumberland, England that graze year-round within a 130-hectare enclosed area is only c. 3‰^25^. Comparative archaeological data from the Netherlands underscore the broad variability in *δ*^18^O values observed in cattle remains. At the Neolithic site of Schipluiden (c. 3600–3300 BCE), δ^18^O_PDB_ values in cattle range from − 6.7‰ to − 1.9‰, yielding a total span of approximately 4.8‰, mirroring the range observed at Swifterbant^30^. At the Iron Age-Roman period site Houten Castellum, cattle *δ*¹⁸O_VSMOW_ values span from 22.7‰ to 28.2‰, which corresponds to an estimated *δ*^18^O_PDB_ range of − 8.0‰ to − 2.6‰ (total range ≈ 5.4‰) ^63^. These consistent ranges across distinct temporal and environmental settings suggest that substantial isotopic variation in prehistoric Dutch cattle was not anomalous but may instead reflect long-standing practices such as the use of diverse water sources, seasonal grazing strategies, or the movement and exchange of animals across regions^26^.

An alternative explanation for the wide *δ*^18^O range in at least group 1 could lie in the environmental variability of the local landscape. In dynamic settings such as the Swifterbant wetlands where there is regular flooding and a mosaic of freshwater sources, livestock may have accessed water from multiple environmental zones, resulting in variable *δ*^18^O values^64^. The mixing of distinct waterbodies within this wetland context could thus contribute to the observed isotopic diversity. However, this explanation is less convincing for sites like Schipluiden and Houten Castellum, which were situated in more stable hydrological settings and yet exhibit similarly broad *δ*^18^O ranges. This raises the possibility that such variation in *δ*^18^O values is a broader characteristic of Dutch (pre)historic livestock, or that mobility among these animals has been underestimated by ^87^Sr/^86^Sr analyses alone.

Supporting the latter interpretation, the strontium isotope data from Swifterbant include one clearly non-local pig (EDAN157, ^87^Sr/^86^Sr = 0.710326), but another pig (EDAN177) stands out for its markedly elevated *δ*^18^O value (–3.8‰), despite having an ^87^Sr/^86^Sr that is compatible with the local signature. Given that seasonal effects on pig molar enamel *δ*^18^O values are typically limited^26,65^, this may reflect a non-local origin not captured by strontium isotopes alone. The oxygen isotope data may therefore provide additional, and possibly more sensitive, evidence for diversity in livestock provenance at Swifterbant. However, further research, including modern comparative datasets from estuarine wetland environments, is necessary to refine interpretations of *δ*^18^O variability in such settings.

The intra-individual *δ*^18^O ranges are relatively modest. The widest observed range among domestic cattle is 2.2‰ (e.g., EDAN191 and 375), which aligns with expected seasonal fluctuations in *δ*^18^O associated with changes in temperature, water intake, and fodder sources^66^.

The *δ*^13^C values from enamel bioapatite provide intriguing additional information on the diet of the group 2 cattle, the ‘forest group’ (Fig. 5). Their *δ*^13^C values range from − 12.8‰ to − 9.6‰. This does not correspond with the forest-based diet seen in their *δ*^13^C_col_ values. When adjusted for a diet-to-enamel enrichment factor of + 14.5‰^67^, even the lowest *δ*^13^C_PDB_ values (–12.8‰; cattle EDAN379) correspond to plant *δ*^13^C values of − 27.3‰ to − 24.1‰. This does not meet the proposed cut-off for canopy effect (–27.5‰)^68^. This could have one of two explanations. It could be a difference in pasture types between early life (as reflected by enamel) and later life (as reflected by collagen). If the animals were kept in open environments as calves, for example close to the settlement, and later pastured as adults in the forest, this could explain this discrepancy. Alternatively, it could be caused by a difference in the source of the cattle’s whole diet versus protein intake. Collagen reflects the isotopic composition of dietary protein, whereas enamel integrates carbon from all macronutrients^69,70^. Twigs, (young) tree leaves, and forbs are more protein-rich than grasses and cattle will selectively forage on these vegetation types to meet their protein requirements^71–73^. If the group 2 cattle were primarily grazing on open grasslands but selectively or seasonally foraged on protein-rich (forest) vegetation or were occasionally (e.g. seasonally in the winter) foddered with such vegetation, their *δ*^13^C_col_ values may be affected without affecting the isotopic composition of the whole diet. Leaf-foddering ruminants has been documented frequently in prehistoric and historic contexts and at the Swifterbant culture site of ‘Doel sector M’ in Belgium, convincing evidence was found for the collection of leaf fodder for livestock^48^. It is currently challenging to disentangle the two explanations to clarify group 2’s *δ*^13^C_PDB_ values. However, in either case, it would appear that management practices were more complex than either pasturing cattle on grasslands or pasturing them in forests.

In the group that is believed to have grazed in the floodplains, cattle *δ*^13^C_PDB_ values range from − 12.3‰ to − 9.0‰, and sheep from − 12.6‰ to − 9.2‰. Combined with their *δ*^13^C_col_ values, this supports the interpretation that these animals grazed in open environments year-round.

The largest individual *δ*^13^C_PDB_ amplitude is seen in cattle EDAN379 (group 2) whose values span from − 12.8‰ to − 9.6‰. These ranges are consistent with seasonal variation in plant *δ*^13^C values and align with those reported for other European Neolithic domestic cattle populations^30,53,74^.

### Implications for the character of Early Neolithic animal husbandry

The results indicate extensive movement and dietary management of livestock at the very onset of the Neolithic in the Dutch wetlands. This finding provides new insights into contact networks and animal management strategies during this period, with broader implications for our understanding of Early Neolithic lifeways in northern Europe. Overall, the new isotope data support the dietary patterns inferred for the animals and suggest a clear distinction between two groups: group one appears to have been primarily composed of animals raised locally (with the exception of EDAN283), while the second group was brought to Swifterbant from (semi-)forested regions elsewhere.

Determining the precise origins of these non-local animals remains challenging, but the ^87^Sr/^86^Sr data and archaeological evidence offer some geographic constraints. Values of between 0.7095 and 0.7110 have been documented relatively nearby, for example in the Pleistocene sandy soils and moraines of the modern Veluwe region (Fig. 1), approximately 40 km from Swifterbant^58^, as well as in the boulder clay areas of the northern Netherlands (unpublished data). These ranges are consistent with the non-local values recorded for cattle EDAN283, 377 (CEJ measurement), 378, 379, and 380, as well as pig EDAN157. However, similar isotopic ratios have also been observed in more distant regions, including areas near the modern Dutch-German border (over 100 km away) and in the southern provinces of North-Brabant and Limburg, more than 150 km south of Swifterbant^58,75^.

The Veluwe and Swifterbant regions were linked geographically in this period by a small regional river system. The upper course of this system corresponds to the present-day river IJssel, while its lower section has since been buried beneath younger sediment deposits. Given the extensive forest cover in the region during the Neolithic, it is plausible that this river corridor served as a primary route for transport and communication. Evidence for human activity in the Veluwe during this period is limited, largely due to two factors. First, preservation conditions in this relatively elevated landscape are poor, resulting in the survival of only durable materials such as flint and stone. Second, there is a lack of diagnostic artefact types that can be securely dated to the late 5th millennium BCE. Nevertheless, the discovery of five perforated wedges (*Breitkeile*) provides the strongest indication of human presence in the Veluwe during this period^76^. These tools are generally dated to between 5000 and 3500 BC^76,77^, and their recovery from settlements at Swifterbant S3^22^ and Tiel^78^ testifies to their importance to the period of relevance here^79^.

Another plausible region of contact lies further to the southeast, in the German Rhineland, an area that would have been accessible via the same interconnected river networks. During the late 5th millennium BCE, this region was inhabited by farming communities associated with the Bischheim culture and its successor, the Michelsberg culture^80^. Strontium isotope analyses of human remains from Bischheim sites have yielded ^87^Sr/^86^Sr ranging between 0.7115 and 0.7128. Although a local baseline for bioavailable strontium has yet to be fully established, faunal data from the same sites, specifically from pigs and deer, show Sr isotope ratios of 0.7106 and 0.7112, respectively. These radiogenic ratios are notably similar to those observed in several Swifterbant cattle. In light of both the isotopic affinities and the riverine corridors that could have facilitated movement, the German Rhineland emerges as a credible candidate for the origin of some of the non-local livestock.

While the precise source areas of the diverse non-local ^87^Sr/^86^Sr cannot be definitively determined, the variation among them clearly indicates that the animals did not all originate from a single location. Furthermore, the intra-elemental data suggest that several cattle moved more than once during their lifetimes. Taken together, these findings provide direct evidence for extensive and complex patterns of livestock mobility in the Early Neolithic. This represents the earliest known instance of such mobility in Europe north of the LBK zone, predating comparable evidence from TRB contexts by several centuries^32,33^.

### Implications for the Neolithisation process

This study emphatically indicates that Swifterbant communities not only kept livestock, a subject of recent debate^81^, but also engaged in highly complex forms of animal management that involved long-distance movement of animals and the integration of herds from diverse origins. The presence of artefacts such as perforated wedges at multiple Swifterbant culture sites underscores the extensive networks of contact during this period. Our findings suggest that livestock were actively embedded in these networks, not only as a means of maintaining genetically healthy herds through breeding, but likely as part of broader socio-economic exchanges^24^.

Moreover, the mobility of livestock implies parallel movements of people. This is supported by the find of one non-local human at the nearby site of Swifterbant S2 (individual S2 900016)^19^. This person (the sex is unknown) has an ^87^Sr/^86^Sr of 0.710649, comparable with two of the cattle and one pig, suggesting possibly the same origin. The presence of non-local cattle, pigs, and humans could reflect groups or individuals travelling to Swifterbant with their animals, merging with or visiting local communities, or gathering for gift exchanges or feasts, such as in later Neolithic periods^26^. Intriguingly, recent genetic research indicates that women at Swifterbant sites often carried Early European Farmers ancestry, while men retained Western European hunter-gatherer lineages^16^. This new evidence for animal mobility therefore supports a plausible scenario in which people, perhaps primarily women, from farming communities joined hunter-gatherer groups, bringing with them livestock and knowledge of animal husbandry. This interpretation has significant implications for how we understand the Neolithisation process in northern Europe and highlights the need for further integrated isotopic and genetic research on both humans and animals.

### Human-cattle relationships

This study further indicates that 4240 BCE marks the onset of increasingly intensive human–cattle relationships in Dutch prehistory. Prior to 4240 BCE, there is scant evidence that Swifterbant communities acquired domestic cattle – unlike pigs, which appear earlier – nor is there much indication of aurochs hunting. Instead, wild boar dominates the faunal record^82^. Between 4240 and 4050 BCE, however, communities were not only keeping cattle but also actively managing their diets and moving them across regions. Still, suids continue to dominate the faunal assemblage (57% of NISP) in comparison to cattle (8%), indicating that subsistence was not yet centred on cattle^10^. Many of the cattle lived well beyond the prime meat production age, reaching the age of three, some even six to eight, while there is no evidence for dairying during this phase^10,44^. This longevity suggests that cattle may have played a primarily social or symbolic role rather than a strictly economic one. By 3600 BCE, at Schipluiden, cattle had become the dominant mammal species (42%) and there is clear evidence for the management of birth seasonality for dairy production^30,83^. By the Bronze Age, cattle represent up to 80% of faunal remains, signalling their consolidation as a central socio-economic resource^24^. While this shift correlates with a gradual decline in the importance of pigs, the involvement of suids in Early Neolithic mobility networks underscores their social and economic significance during this transformative period.

### Considerations for future research

Given the cost and labour intensity of isotopic analyses, strategic sampling decisions are essential. In this study, we aimed to balance breadth and depth by analysing as many individuals as possible (for cattle and caprines, all available dental elements) to assess mobility patterns at a population level, while including additional samples to explore individual movement histories. This represents a middle ground between two common approaches in mobility studies: sampling a single tooth per animal across many individuals to evaluate broad-scale trends, or sequential sampling of a few individuals to investigate seasonal mobility, diet, and birth timing. Future studies should also include phosphate oxygen isotope (*δ*^18^Oₚ) analyses, allowing comparison with *δ*^18^O_PDB_ values to assess enamel preservation and strengthen interpretations. While sequential sampling yields rich information about individuals, it often limits insights into broader herd-level patterns.

Our strategy has proven effective in revealing both population-level patterns and intra-individual mobility in the Swifterbant livestock. The data do not allow us to subsequently determine whether this mobility was intermittent or seasonal, which would indicate transhumance practices. A logical next step would be to sequentially sample the non-local animals from Swifterbant to further investigate the nature of livestock and human mobility in the Early Neolithic.

## Conclusion

This study provides the earliest evidence for livestock mobility in the Neolithic north of the LBK zone in Europe. At Swifterbant, five non-local cattle and one non-local pig were identified, while no non-local sheep were identified. The strontium isotope variation among these individuals indicates that they originated from multiple distinct regions, rather than a single source. Moreover, four cattle show isotopic ratios consistent with movement across different geological zones during early life, suggesting that livestock were relocated multiple times before arriving at Swifterbant. These findings highlight the importance of analysing both intra-individual movement and broader population-level trends to fully understand the scale and complexity of livestock mobility.

Except for one individual, the mobility patterns align with previously observed dietary differences among the livestock. One group of cattle and sheep appears to have been raised locally, or in geologically similar areas influenced by varied water sources, with one clear non-local exception. In their later life, these animals were all kept at Swifterbant, grazing in open meadows under the influence of regular flooding. The other group consisted of animals that lived in different locations during their early life, as adults were pastured in (semi-)forested environments or foddered with protein-rich (forest) vegetation, and eventually brought to Swifterbant.

The results indicate that in the Dutch Early Neolithic, at the earliest demonstrated onset of farming, livestock husbandry involved sophisticated practices coupled with far-reaching mobility and exchange networks. This can altogether be seen as the beginning of intensive human-cattle relationships in Dutch prehistory. The livestock mobility also serves as a valuable proxy for human movement in a period where direct evidence for human mobility remains sparse. Considering recent genetic findings showing women with Early European Farmers ancestry joining Swifterbant communities, it is plausible that these movements reflect women migrating with their animals and expertise, actively contributing to the spread of farming knowledge and practices in the region.

## Materials and methods

### Sample selection

In a previous study, collagen from the bones and dentine of cattle, suids, and caprines from the Swifterbant sites S3, S4, and Windplan Blauw was analysed for carbon and nitrogen isotopes. For the current study, due to the lack of dental elements from Windplan Blauw, only (pre)molar enamel from S3 and S4 was sampled for combined strontium, oxygen, and carbon isotope analysis. Given the limited number of suitable teeth from S3 and S4, the sampling strategy was adjusted to maximize the dataset, including not only previously analysed individuals but also all suitable cattle and sheep (pre)molars from these sites. Consequently, a total of 20 cattle (*Bos* sp.), six suids (*Sus sp*.: *Sus domesticus/Sus scrofa*), and two sheep (*Ovis aries*) teeth were selected and sampled, including lower and upper dP4, P4, M1, M2, M3, and M1/M2 (Table S1). Although some individuals may be represented twice among the samples, the variation in collagen and enamel ^87^Sr/^86^Sr, *δ*^18^O, *δ*^13^C, and *δ*^15^N values suggests a high number of distinguishable individuals. For one cattle individual (EDAN193) and one sheep (EDAN352), both the M1 and M2 were sampled.

We argue that it is highly likely that all of the *Bos* teeth belong to domestic cattle (*Bos taurus*) based on the following evidence:

i) Postcranial osteometric analysis shows that the *Bos* assemblage at S3 includes cattle significantly smaller than LBK domestic cattle, except for one specimen identified as an aurochs (*Bos primigenius*) due to its larger size;
ii) The measurements of the sampled teeth are congruent with the size of domestic cattle^10^;
iii) The collagen *δ*^13^C and *δ*^15^N values indicate that all specimens with elevated *δ*^15^N have a diet inconsistent with that of aurochs^10,84,85^.

The sheep were previously identified from goats through Zooarchaeology by Mass Spectrometry^10^. The suid teeth were selected from specimens most likely to be domestic pigs, based on their size and diet^10^.

### Sample preparation

One sample was taken from each suid tooth. Intra-tooth sampling was conducted for cattle and sheep where possible. Since cattle and sheep teeth grow incrementally, the location of each sample provides information on the animal’s mobility at different ages (Table 3). A location near the apex of the tooth, representing the earliest preserved enamel (the apex itself is commonly worn), the cemento-enamel junction (CEJ), which mineralizes last, and the midway point between these two locations on the enamel (referred to as the mid-enamel sample) were sampled. Mid-enamel samples were analysed only for specimens whose near apex and CEJ isotope ratios showed significantly different results. Sequential sampling for seasonality investigation was not performed to maintain cost and time efficiency. Sampling up to three enamel locations per tooth allowed for a more comprehensive analysis of the available assemblage rather than focusing on only a few individuals. The material remains available for further sequential sampling if needed.

Depending on the physical quality of the enamel, the buccal or lingual lob of the molars was mechanically cleaned through abrasion using a Dremel drill equipped with a diamond-tipped ball burr. Approximately 10 mg of crisp white enamel powder was sampled and sealed in a 2 ml polyethylene Eppendorf^®^ centrifuge tube. The burr tip was cleaned with Milli-Q, 10% HCl, Milli-Q, and ethanol after every sample to avoid cross contamination. Circa 2 ± 1 mg was subsampled in a 3 M HCl cleaned Eppendorf^®^ tube and transferred to the ISO 5 clean laboratory with ISO 4 laminar flow hoods at the Vrije Universiteit Amsterdam. Without any further pretreatment of the samples to remove secondary carbonates^86^, 0.3 mg ± 10% was subsampled in clean screw capped Exetainer^®^ vials and transferred to the Stable Isotope Laboratory at the Vrije Universiteit Amsterdam for O-C isotope analysis.

A detailed description of the column extraction and the sample loading procedures for the strontium isotope analyses is given elsewhere^87^. The isotope compositions were measured using a Thermo Scientific™ Triton Plus™ instrument. The ^87^Sr/^86^Sr were determined using a static routine and were corrected for mass fractionation to ^86^Sr/^88^Sr of 0.1194. The reproducibility was 0.710258 ± 0.000007 for repeated analysis of NIST^®^ SRM^®^ 987 standard during the study (2023, *n* = 102, 1s, loading size 200 ng). The total procedural blanks (*n* = 3) contained between a negligible amount of strontium. The ^87^Sr/^86^Sr are reported (Table 1) plus minus two standard error (2SE), representing the analytical uncertainty calculated from 240 measurements (12 blocks of 20 cycles) within each run. The ^87^Sr/^86^Sr are compared to the ^87^Sr/^86^Sr isoscape reported in Kootker et al. 2016^58^ and new baseline ^87^Sr/^86^Sr data^88^. These ^87^Sr/^86^Sr data are based on archaeological rodents and modern vegetation with different rooting depths (i.e., grasses, shrubs, and trees) from regions with minimal anthropogenic influence.

The *δ*^18^O and *δ*^13^C were measured using a Thermo Finnigan GasBench II preparation device interfaced with a Thermo Finnigan Delta+ mass spectrometer. The Exetainer vials containing the enamel samples were placed in a heated aluminium tray maintained at 70 °C. The autosampler automatically injected 100% anhydrous phosphoric acid into each vial, after which the samples were allowed to react for 1.5 h prior to isotopic analysis. The data were normalised to the Vienna Peedee Belemnite (VPDB) scale using an in-house carbonate reference material (VICS) calibrated against NBS19 (RM 8544) and LSVEC certified reference materials. The international control standard IAEA-603 was used to check instrument performance (average 2.49 and − 2.37‰ for *δ*^13^C and *δ*^18^O respectively, *n* = 15). The reproducibility of IAEA-603 during the analytical session was 0.11‰ and 0.17‰ for *δ*^18^O and *δ*^13^C respectively (1s).

## Supplementary Information

Below is the link to the electronic supplementary material.

Supplementary Material 1

Supplementary Material 2

## Data Availability

All data supporting this study are available within the online supplementary materials. All background data are available in the online supplementary materials of Brusgaard et al. 2024^10^. The isotope data are openly available as a dataset on IsoArcH.org: https://doi.org/10.48530/isoarch.2026.009^89,90^.
