# Supplementary material for: Isotope analysis reveals mobility of the earliest cattle herds in northern Europe
Source: Sci Rep. 2026 Sep 6;16:35619. doi: 10.1038/s41598-026-66057-4 (PMC13723414; doi:10.1038/s41598-026-66057-4)
Supplement: Supplementary file 1 [file 41598_2026_66057_MOESM1_ESM.docx]

Figure S1. Bivariate plots showing intra-individual results of ^87^Sr/^86^Sr versus *δ*^18^O for Swifterbant cattle and sheep. Background shading indicates the local ^87^Sr/^86^Sr range (dark grey: 0.7088–0.7092) and the maximum regionally available ^87^Sr/^86^Sr range (light grey: 0.7092–0.7095).

Figure S2. Bivariate plots showing intra-individual results of *δ*^13^C versus *δ*^18^O for Swifterbant cattle and sheep.
